# Changes in Smoking Behaviors following Exposure to Health Shocks in China

**DOI:** 10.3390/ijerph15122905

**Published:** 2018-12-19

**Authors:** Qing Wang, John A. Rizzo, Hai Fang

**Affiliations:** 1 School of Public Health, Shandong University, Jinan 250012, China; wangqing1984@126.com; 2 Department of Family, Population & Preventive Medicine, State University of New York at Stony Brook, Stony Brook, NY 11794, USA; john.rizzo@stonybrook.edu; 3 China Center for Health Development Studies, Health Science Center, Peking University, Beijing 100083, China

**Keywords:** health shocks, spousal, smoking, China

## Abstract

*Background*: Evidence suggests that following major individual health shocks, smokers change their smoking behaviors. However, little is known about the association between spousal health shocks and smoking. This study examined the contemporaneous and long-term effects of individual and spousal health shocks on males’ smoking behaviors in China. *Methods*: This study employed a nation-wide data base from the 1991–2011 China Health and Nutrition Study. Random effects models were estimated to ascertain the impacts of health shocks on males’ smoking behavior. Smoking behaviors were measured by smoking status, smoking consumption and smoking cessation. *Results*: In the short term, respondents who incurred health shocks decreased their likelihood of smoking by 10%. In addition, health shocks decreased the likelihood of heavy smoking versus the combined moderate and light categories by 41.6%, and increased their likelihood of quitting by 85.3% for ever smokers. Spousal health shocks had no significant effects on individual smoking behaviors. The long-term effects were similar to the short term impacts. *Conclusions*: People changed their smoking behaviors in response to their own health experiences but not those of their spouses. Antismoking messages about the health effects on others are unlikely to influence individual smoking behaviors, unless individuals believed that they are personally vulnerable to smoking-related diseases.

## 1. Introduction

The tobacco epidemic has now assumed pandemic proportions, with approximately 1.3 billion tobacco users and 6 million annual deaths from tobacco use. The pandemic also involves substantial healthcare, social, and economic costs across high-, middle-, and low-income countries [1]. Smoking in China is prevalent, as the People’s Republic of China is the world’s largest consumer and producer of tobacco. China has one-third of the world’s smokers, and more than 700 million people regularly exposed to second-hand smoke. One million people die from tobacco-related disease each year. Of these, 100,000 deaths were due to exposure to second-hand smoke [2,3]. In view of the high health risks resulting from smoking in China, tobacco use causes many heads of household (especially in low-income families) to incur death or disability, impoverishing these families [4].

The Chinese government committed to achieving a smoke free-indoor environment when the WHO Framework Convention Tobacco Control was signed in 2006. Nevertheless, there remained a large gap between the WHO Framework Convention Tobacco Control requirements and smoking in China. [5]. Tobacco control in China remained particularly ineffective due to incomplete information about the true adverse effects of smoking partly due to interference by the tobacco industry [6].

Smoking can be viewed as a divestment in the accumulation of health capital in return for a short-term increase in utility [7]. A well-accepted assumption implies that individuals are attracted to risky behaviors, when they believe that the gain outweighs the loss due to lack of information on smoking risk. Providing complete information on health risk is an important lifestyle intervention to influence behavioral changes. 

There is evidence that, following major individual health shocks, smokers do change their smoking behaviors [8,9,10]. However, when examining the impacts of health shocks to others (e.g., parental, spousal or neighbors’ health shocks), the results vary greatly [11,12,13]. A study of adult Chinese men observed that learning of a neighbor’s lung cancer diagnosis substantially affected decisions about continued smoking and intentions to quit, whereas another study estimated the effects of parental health shocks on adult offspring smoking behaviors in United States and found limited evidence that offspring smoking behaviors were sensitive to parental health [11,13]. 

Do spousal health shocks affect smoking behavior? Marriage is a uniquely close relationship in which two persons share feelings, information and values [14]. The partners develop a verbal and particularly non-verbal communication system (posture, gesture) that increases their possibilities of interaction in order to achieve marital satisfaction [15]. Thus, a couple’s interaction could be an important information sources for individuals to update subjective beliefs about smoking’ harms [12]. When the smoker lives with a spouse who has a health shock, he or she will get a chance to learn about the potential health hazard of smoking including second-hand smoking, which may lead them to update their subjective beliefs and potentially change their smoking behaviors. However, many factors such as a change in work and sex role patterns following a health shock may hinder a couple’s effective interaction. 

The question of whether spousal health shocks are associated with smoking behaviors remains unanswered in China. Indeed, to our knowledge there is only one study on the effects of spousal health shocks on smoking behaviors. Using US data on a panel of individuals from the Health and Retirement Study of persons aged 51 to 61 in 1992, the contemporaneous effects of spousal health shocks among older persons were analyzed, finding that they did not change their smoking behaviors in response to information about spousal health shocks [12]. However, it might take some time for smokers to deal with health shocks and change their smoking behaviors [16]. Moreover, older persons have different time preferences from younger individuals, so these findings should be generalized to the entire population with caution. The present study employed data from China to investigate individual and spousal health shocks on smoking behaviors in short- and long-term periods for a general population of male adults. We use data from China Health and Nutrition Survey (CHNS) for males aged 18 to 101 over the years of 1991–2011. The prevalence of adult smoking was 52.1% for males and 2.7% for females in 2013 [4]. Since the vast majority of smokers in China are males, we focused on males’ smoking behaviors. The association of health shocks and an individual’s smoking behavior is critical for anti-smoking campaigns because it provides insight into the potential influence of anti-smoking interventions. Thus, this study helps inform policy makers in their efforts to reduce smoking in China.

## 2. Methods

### 2.1. Participants

We used data from the 1991–2011 China Health and Nutrition Study (CHNS) [17]. The survey was designed to examine the effects of the health, nutrition, and family planning programs implemented by national and local governments in China and to examine how the social and economic transformation of Chinese society was affecting the health and nutritional status of the Chinese population. CHNS was a longitudinal survey that covered 9 provinces with substantial variations in geography, economic development, public resources, and health indicators in 1991, 1993, 1997, 2000, 2004, 2006, 2009 and 2011. The nine provinces accounted for approximately 45% of China’s population in 2011. The surveys collected data on consumer goods as well as detailed information on measures of health outcomes, such as height, weight, blood pressure, activities of daily living, self-reported health status, morbidity, physical function limitations, and disease history. This study used secondary data of CHNS, and we are exempt of ethical approval. CHNS has initially been approved by the institutional review committees of the University of North Carolina at Chapel Hill, the National Institute of Nutrition and Food Safety, Chinese Center for Disease Control and Prevention, and the China-Japan Friendship Hospital, Ministry of Health.

A multistage, random cluster process was used to draw the sample surveyed in each of the provinces. Counties in the nine provinces were stratified by income levels (low, middle, and high) and a weighted sampling scheme was used to randomly select four counties (one in low, two in middle, and one in high-income levels) from each province. In addition, the provincial capital and the lowest-income city were often selected. Villages and townships within the counties and urban and suburban neighborhoods within the cities were selected randomly. There were approximately 4400 households in the 2011 survey. 

After excluding missing data, the final sample size for analysis included 21,942 individuals. In addition, a subsample consisting of persons who ever smoked was also used. An ever smoker at any wave is defined as someone who smoked before or as of that wave (N = 13,914). 

### 2.2. Measures

The first indicator measuring smoking behaviors was current smoking status. Smoking status was classified as current smoker or non-smoker in the survey year. Current smokers referred to respondents who smoked at the time of the interview, and were coded as 1. 

Ever smokers were asked follow-up questions on smoking cessation and smoking consumption. If respondents smoked before but stopped smoking during the current survey period, they were regarded as having quit smoking and encoded as 1. Smoking consumption was assessed by the number of cigarettes smoked daily. This variable was recoded into an ordinal variable from 1 to 3 representing the level of smoking (1 = light smokers at present (0–10 cigarettes); 2 = moderate smokers (11–20 cigarettes); 3 = heavy smokers (21 or more cigarettes)) [18,19]. The groups of light smokers, moderate smokers and heavy smokers may indicate a different responsiveness to environmental cues that light smokers are affected more by environment [20]. 

Health shocks consisted of adverse health events found to have an elevated relative risk for death [9]. Using CHNS data from each wave from 1991 to 2011, health shocks in our study include high blood pressure, diabetes, myocardial infarction, lung cancer, asthma, and stroke or transient ischemic attack. In one way or other, smoking or second-hand smoking might cause such events [21,22,23]. In our study, health shock variables were defined as experiencing any of the above events between two survey waves and they represented onsets of new and serious health conditions. For example, if a respondent reported a heart attack in one wave, and no history of heart attack in the previous waves, this was recorded as a health shock in that wave. We constructed the health shock variables in terms of onset between the current and the previous waves to minimize reverse causality of smoking on health shocks. Such health shocks might motivate respondents to obtain information on the health risks of smoking that may affect their beliefs about survival probabilities. Health shocks were coded into four categories: no health shocks to both the individual and his spouse (reference group); health shocks to spouse only; health shocks to individual only; and health shocks to both.

Other control variables included a variety of demographic factors. Age was measured as a continuous variable, and a log transformation was used in regression models to correct for right-skewness. Educational attainment was defined at three levels: “primary school or below”, “junior high school”, and “senior high school or above”. Two binary variables for educational attainment were constructed, with primary school or below serving as the reference group. Job status was also measured as a binary variable, using unemployed as the reference group. Another binary variable was included to capture whether or not the individual resided in an urban area. Household income per capita was also controlled for. We converted household income per capita to constant 2011 Chinese Yuan using the Consumer Price Index. 

We also included a variable measuring drinking behavior. Previous studies suggested that smoking and drinking were interdependent, thus the question of whether smoking and drinking were complements or substitutes was of crucial interest to determine the effects of health shocks [24]. Drinking was found to be complementary to smoking, which was supported in several studies using data from China [25,26,27]. If health shocks lead to a reduction in drinking and smoking, failure to control for drinking behavior will lead to an overestimate of the effects of health shocks in terms of reducing smoking. Drinking behavior was determined by responses to the following survey question: “How often did you drink beer or any alcoholic beverage?” “Almost every day and 3-4 times a week” was coded as a frequent drinker; “once or twice a week” was regarded as a less frequent drinker; “once or twice a month” was coded a very light drinker; and “no more than once a month and never drink” was coded as non-drinker (the reference group). Provinces where the respondent resided and year-specific fixed effects were also included to help control for unobserved factors that might affect the outcomes of interest.

### 2.3. Statistical Analysis

Both static and dynamic models were employed in this analysis. The static model included contemporaneous effects only, while the dynamic model allowed for lagged effects of health shocks on smoking behaviors. In a static context, a random effects model was applied. Smoking status was regressed on all the male respondents. Since smoking status is a binary variable, we used individual random effects logistic regression to analyze the relationship between smoking and health shocks from self and/or spouse. Among those ever-smokers, the effects of health shocks on smoking cessation and smoking consumption were then estimated. For smoking cessation, individual random logistic effects models were employed; for smoking consumption classified into light smokers, moderate smokers and heavy smokers, an individual random ordered logistic effects model was used. Odd Ratios (OR) and 95% confidence interval (CI) were reported for individual random effects logistic regression. At the same time, cigarettes per day, as a continuous variable, was estimated by an individual random model. Results from this model were consistent with those from an individual random ordered logistic effects model. Marginal effects were also reported. Stepwise modeling with forward selection was used to determine control variables. Stepwise modeling began with health shocks, then demographic, socioeconomic circumstances and health behavior variables were added. T test was applied to test the addition of each variable. Finally, log of age, education, household income, and drinking behavior were adjusted.

A dynamic econometric model was applied to evaluate a lagged effect of health shocks on smoking behavior. Due to the delayed response to health shocks, the effects of health shocks on smoking were expected to occur after the onset of health shocks. Thus, the infinite distributed lag (IDL) model was used to allow for lagged effects. IDL models, widely utilized in economic studies, provide a systematic way to investigate the distribution of effects over time. In the IDL model, the smoking behavior of person in the previous survey wave were also controlled for, so that no lag length needed to be taken into account for lagged effect of health shocks on smoking. The IDL model can estimate the delayed and cumulative effects of health shocks on smoking behaviors over the entire lag period simultaneously. Generalized Method of Moments was applied to estimate the IDL model with individual health shocks were treated as a predetermined variable, which could solve the potential endogenous issue because of measure errors. The marginal effects of health shocks on smoking behaviors between two successive waves of data measured the short-term effects of health shocks. When the absolute value of the lagged smoking behavior’ marginal effects is less than one, the long-term effects were calculated by the ratio of the short-term effects of health shocks to one minus the marginal effects of lagged smoking behaviors, which considering the influence of health shocks in close to infinite time (long_term_effect = short_term_effect/(1-lagged_marginal_effect) [28]. Marginal effects were calculated for IDL model. The standard errors were robust to autocorrelation as well as heteroscedasticity. All analyses were performed using STATA version 14.0 (StataCorp, College Station, TX, USA). 

## 3. Results

Table 1 shows the descriptive summary of variables for the entire sample. Approximately 65 percent of respondents reported that they smoked before, and 59 percent of respondents were current smokers at the survey time. The distribution of smoking in the CHNS data was very similar to those in the Global Adult Tobacco Survey China 2010 [2].

On average, ever-smokers consumed 14.96 cigarettes daily. Among them, 47.95 percent were moderate smokers (smoked 11–20 cigarettes daily) and 11.02 percent heavy smokers (smoked 21 or more cigarettes daily), while 8.59 percent of male ever-smokers had quit smoking. Approximately 5.89 percent of respondents reported having their own and/or spousal smoking-related health shocks between two waves. Among them, 3.18 percent of respondents reported his own health shocks; 2.27 percent of respondents reported spousal health shocks; 0.44 percent of respondents reported both himself and spousal health shocks. Health shocks were mostly caused by high blood pressure and diabetes. 60% of health shocks were related to high blood pressure. High blood pressure issue is a common health risk in China, and continues to be a serious problem. Around 34% Chinese suffered from hypertension in 2010 [29]. Previous research has supported that smoking is a major independent risk factor of the high prevalence of high blood pressure [30].

Current smokers referred to respondents who smoked at the time of the interview, and was encoded as 1. If respondents smoked in the past but stopped smoking now, respondents at this survey time are regarded as quitters and encoded as 1. Smoking consumption was recoded into an ordinal variable from 1 to 3 representing the level of smoking (1 = light smokers at present (0–10 cigarettes); 2 = moderate smokers (11–20 cigarettes); 3 = heavy smokers (21 or more cigarettes)). Health shocks were mostly caused by high blood pressure and diabetes. 60% of health shocks were related to high blood pressure.

**Figure 1:**
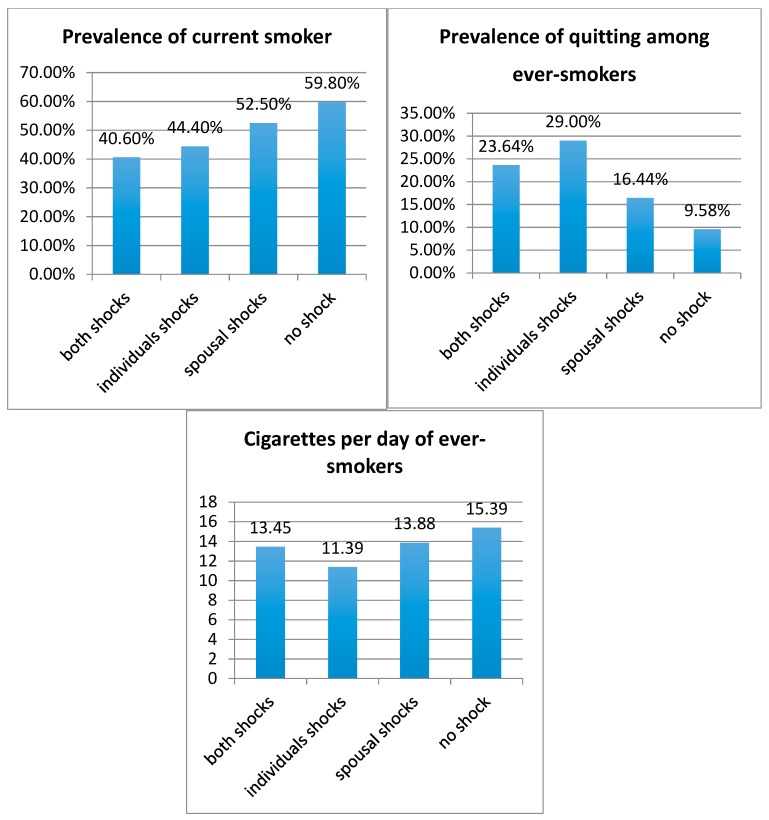
Relationship between health shocks and smoking behaviors

Figure 1 reveals a negative association between health shocks and smoking behaviors. Compared to those exposed to no health shocks, respondents who suffered from health shocks or whose spouse incurred health shocks had a lower prevalence of current smoking, and ever-smokers had smoked fewer cigarettes per day and an increased rate of quitting smoking.

Table 2 shows individual random effects logistic regression results for the effects of health shocks on smoking behaviors, after controlling for socioeconomic status, drinking behaviors and other demographic characteristics. Individual health shocks had statistically significant effects on smoking behaviors (*p* < 0.01); however, spousal health shocks had no significant effects. 

Table 3 presents the marginal effects of health shocks on smoking behaviors among China’s male population. Individual health shocks decreased the likelihood of smoking by 10% (*p* < 0.01). Individual health shocks also decreased smoking consumption and increased the quit rate among ever smokers. More specifically, individual health shocks decreased the likelihood of heavy smokers versus the combined medial and light categories by 41.6% (*p* < 0.05), and increased their likelihood of quitting by 85.3% (*p* < 0.01) among ever smokers. In contrast, the spousal health shocks alone had no significant effects on smoking behaviors. 

Table 4 presents the short-term and long-term marginal effects of health shocks on smoking behavior using the IDL model. In the dynamic model, the results were consistent with those in the static framework. 

Individual health shocks were significantly associated with reduced smoking behaviors in the short-term period. In addition, the long-term and short-term marginal effects were very similar.

## 4. Discussion

Using data from CHNS 1991–2011, this study examined the effects of individual and spousal health shocks on smoking behaviors among China’s male population. If smokers learned about tobacco risk as the result of these shocks, one might expect to observe changes in smoking decisions [31]. On the other hand, if there was no learning, then no changes in smoking behaviors may be observed. 

Around 6% of our sample experienced smoking-related health shocks. The low level of health shocks might make respondents think that a smoking-related health shock is a low probability event that is unlikely happen to them. Therefore, a low incidence of health shocks may underestimate the effects of health shocks on smoking behaviors. Nevertheless, we found that individual health shocks affected smoking, quitting, and smoking consumption in the short-term and long-term periods, after controlling for a variety of demographic, family background, and other characteristics. However, individuals were not found to change their smoking behaviors in response to spousal health shocks. These results were consistent with the findings about the effects of own health shocks [8,9,10,11,12] and the results evaluating the effects of others’ health shocks on smoking behaviors [11,12]. People’s smoking behavior changed in response to their own health shocks but not those of others. Given the information delivered from health shocks, respondents only change their smoking behaviors when they believe they are vulnerable to smoking-related health risks. In other words, they had to learn from direct adverse experiences, which were construed as “the hard way”.

Communication effectiveness between couples may partly explain the fact that people didn’t learn from spousal health shocks in China. In many Asian countries, including China, traditional sex role patterns persist within the family. For example, males assume primary responsibility for supporting the family financially and females’ responsibility was to do housework and raise children. Overall, females possessed low authority both in the family and society [32]. Now, however, females increasingly participate in the labor market and gain more financial independence and authority within the family [33]. The conflicts between traditional gender stereotypes and changing gender roles may lead to marital conflicts and obstruct effective communication [34]. If an individual does not obtain enough information about the risk of smoking from spousal health risks due to ineffective couples’ commutation, they might not change their smoking behaviors in response to spousal health shocks.

On the other hand, one study estimated the effects of proximity to a lung cancer neighbor on smoking and found that such proximity affected decisions about continued smoking and intentions to quit [13]. One potential explanation of the heterogeneous results might be due to the limitation of our study that health shocks were general ones so that we are not sure smoking is the direct cause of health shocks. Health shocks in this study were mostly caused by high blood pressure and diabetes. In addition, risk is always subjective but in cases in which it is fairly homogeneous across individuals, it is easier to learn from others’ experiences such as, impacts of seatbelts in preventing serious injuries. However, in instances in which there is sufficiently great heterogeneity in risk, individuals seem to attach different levels of credibility to information according to its sources (e.g., personal experiences or experiences of others or general information). The low prevalence of ever smokers for females and health shocks may also contribute to the heterogeneous results. However, what should be noted is that second-hand smoke is a big issue in China. As mentioned earlier, there are 100,000 deaths each year due to exposure to second-hand smoke. Many wives are very likely to expose to second-hand smoking, and suffer from health shocks related to second-hand smoking. 

The long-term effects of health shocks on smoking were quite similar to the short-term results. This reflected that the marginal effects of health shocks on smoking behaviors declined rapidly over time. Smoking reduction and cessation was a difficult process. Studies showed that more than half of all smokers attempted to quit, but only half of these were successful [35,36]. Thus, if one’s health shock did not induce a change in smoking behaviors quickly, it was unlikely to induce changes later. 

This is the first study to quantify the relationships between health shocks and smoking behaviors in China. We also extended the methods for analyzing the association of health shock and smoking behaviors in following ways. In order to control for multi-collinearity of one’s own and spousal health shocks (the variance inflation factor: 6.19), one’s own and spousal health shocks in this paper were replaced with a group of dummy variables: no health shocks to the individual and the spouse; health shocks to spouse only; health shocks to the individual only; and health shocks to both. Second, the long-term effects and the short term effects were compared. We find that the long-term effects and the short-term effects were very similar. Our findings in combination with other studies shed light on the implementation of public policies to reduce smoking. In sum, people learn “the hard way” When people learn about the health hazards of smoking by experiencing a health shock, they change their smoking behavior. This suggests that smoking interventions and antismoking advertisements based on information provision alone may not be very effective, unless the antismoking messages were designed to be highly personalized. Thus, if the goal is to influence the smoking behaviors of a number of major demographic groups, advertisements tailored to each of these groups are more likely to be successful than generic ads. News media often use personal stories to which individual readers could relate, and anti-smoking advertisements have proven to be more successful if they follow this approach. In addition, anti-smoking interventions should ensure that the information on health risk of smoking was up-to-date. Over time, the negative effects of health shocks on smoking behaviors diminish rapidly, thus the long-term effects and the short -term effects were very similar.

This study has some important limitations that must be mentioned. Both smoking behavior and the occurrence of health shocks are self-reported. Since under-reporting of smoking is well reported, our models may underestimate the effects of health shocks on smoking. Additionally, some respondents died and therefore they did not reply to the following survey or surveys, which could lead to attrition bias. However, since many of these individuals probably had a chronic illness for a significant period, their elimination from the panel might not change the basic results.

## 5. Conclusions

Using a nationally-representative data base, this study estimated the effects of individual and spousal health shocks on smoking behaviors among Chinese men. Individual health shocks were related to decreases of smoking behaviors measured by smoking status, smoking consumption and quitting behavior. However, no change in smoking behaviors were observed in response to spousal health shocks. Individuals learn from their own experience, but not from others. Thus, antismoking intervention should be designed to be more personalized.

## Figures and Tables

**Table 1:** Respondents’ socioeconomic and life style characteristics.

Variables	Percent
Health shocks between two survey wave	
No shock (%)	94.11
Individual shock (%)	3.18
Spouse shock (%)	2.27
Individual and spouse shock (%)	0.44
Current smokers (%)	59.00
Quitting smoking for ever-smokers (%)	8.59
Current number of cigarettes smoked daily for ever-smokers Mean (Std. dev.)	14.96 (9.91)
Age Mean (Std. dev.)	52.52 (12.69)
Household size Mean (Std. dev.)	4.08 (1.61)
Urban (%)	33.64
Unemployed (%)	7.23
Household income per capita (Thousand Yuan) Mean (Std. dev.)	8.11 (11.33)
Education level	
Primary school and below (%)	44.61
Secondary school (%)	31.35
High school and above (%)	24.04
Drink behavior	
Drink frequently (%)	23.23
Drink less frequently (%)	24.64
Drink barely (%)	9.86
Non drinker (%)	47.27

**Table 2:** Effects of health shocks on smoking behaviors with individual random effects logistic regression.

	Current Smokers	Quitting	Cigarettes Per Day
Variables	OR (95% CI)	OR (95% CI)	OR (95% CI)
Health shock			
Individual health shock	0.68 **	2.35 **	0.66 **
	(0.52–0.89)	(1.71–3.22)	(0.52–0.85)
Spouse health shock	0.97	0.95	1.24
	(0.72–1.31)	(0.63–1.42)	(0.95–1.62)
Individual and spouse health shock	0.40 *	2.73 *	0.99
	(0.19–0.85)	(1.01–7.35)	(0.49–1.99)
Log of age	0.21 **	53.44 **	0.33 **
	(0.14–0.30)	(31.88–89.58)	(0.26–0.43)
Urban	0.84	1.17	0.67 **
	(0.70–1.01)	(0.96–1.42)	(0.59–0.76)
Employed	1.71 **	0.61 **	1.74 **
	(1.49–1.97)	(0.50–0.74)	(1.54–1.96)
High group of income per capita	0.80 **	1.66 **	1.06
	(0.70–0.91)	(1.38–2.00)	(0.96–1.18)
Education level			
Secondary school	0.77 **	1.03	1.05
	(0.65–0.91)	(0.83–1.27)	(0.92–1.19)
High school and above	0.48 **	1.13	0.75 **
	(0.40–0.59)	(0.88–1.44)	(0.65–0.88)
Drink behavior			
Drink frequently	5.46 **	0.63 **	2.08 **
	(4.69–6.36)	(0.51–0.77)	(1.84–2.35)
Drink less frequently	3.79 **	0.71 **	1.07
	(3.30–4.34)	(0.58–0.88)	(0.96–1.20)
Drink barely	2.43 **	1.13	0.74 **
	(2.04–2.89)	(0.87–1.47)	(0.63–0.86)
Observation	21,942	13,914	13,914

Log of age, education, household income, and drinking behavior were adjusted. * Significant at the 5% level; ** Significant at the 1% level.

**Table 3:** Marginal effects of health shocks on smoking behaviors with individual random effects logistic regression.

Health Shock	Current Smokers	Quitting	Cigarettes Per Day
Individual health shock	−0.100 **	0.853 **	−0.416 *
Spouse health shock	0.016	0.054	0.218
Individual and spouse health shock	−0.145 **	1.004 *	−0.012

Log of age, education, household income, and drinking behavior were adjusted. * Significant at the 5% level; ** Significant at the 1% level.

**Table 4:** Long-term marginal effects of health shocks on smoking behaviors with IDL model.

Variables	Current Smokers		Quitting		Cigarettes Per Day	
	Short−Term	Long−Term	Short−Term	Long−Term	Short−Term	Long−Term
Health shock						
Individual health shock	−0.02	−0.02	0.04 **	0.04 **	−0.16 **	−0.17 **
Spouse health shock	0.00	0.00	−0.02	−0.02	0.08	0.08
Individual and spouse health shock	−0.08 *	−0.08 *	0.10 *	0.10 *	−0.10	−0.11
Lag of smoking status	0.04 **		−0.05 *		0.05	

Log of age, education, household income, and drinking behavior were adjusted. * Significant at the 5% level; ** Significant at the 1% level.

## Abbreviations

CHNS	China Health and Nutrition Study
OR	Odd Ratios
CI	Confidence Interval
IDL	Infinite Distributed Lag
